# Possible Contribution of T-pattern Detection and Analysis to the Study of the Behavioral Correlates of Afferent Inhibition

**DOI:** 10.3390/brainsci10110818

**Published:** 2020-11-04

**Authors:** Maurizio Casarrubea

**Affiliations:** Laboratory of Behavioral Physiology, Department of Biomedicine, Neuroscience and Advanced Diagnostics (Bi.N.D.), Human Physiology Section “Giuseppe Pagano”, University of Palermo, 90134 Palermo, Italy; maurizio.casarrubea@unipa.it

**Keywords:** transcranial magnetic stimulation, afferent inhibition, short-latency afferent inhibition, long-latency afferent inhibition, T-pattern analysis, TPA

## Abstract

A pivotal tenet in modern behavioral sciences is that the study of behavior, in its most intimate structure, necessarily deals with time and, for this reason, behavioral dynamics are not intuitively perceivable and/or detectable (Eibl-Eibesfeldt, 1970). In reality, the possibility to describe a given behavior in terms of its structural/temporal features makes available new and detailed information otherwise unavailable. The aim of the present paper is to discuss the possible application of T-pattern detection and analysis, i.e., a multivariate approach specifically developed to describe the temporal structure of behavior, to the study of an important and still scantly investigated issue, namely the detection of behavioral correlates of the neurophysiological phenomenon known as afferent inhibition.

Introduced more than three decades ago by Barker and colleagues [1], transcranial magnetic stimulation (TMS) is a technique consisting of the generation of an intense magnetic field delivered by a current pulse propagating within a conductive coil placed on the scalp [2]. Following appropriate adjustments of different parameters, such as amplitude, duration and localization of the delivered stimuli, it is possible to activate given brain regions by depolarizing neuronal populations receiving these currents [2,3]. Of course, the observed effect of TMS strictly depends on the cortical area stimulated. TMS currents delivered on specific areas of the primary motor cortex do result in a consistent activation of the corticospinal pathway. The resulting response, in the pertinent muscle, is called motor-evoked potential (MEP) [2,3]. Importantly, differently from transcranial *electrical* stimulation, TMS is totally painless and non-invasive. It goes without saying, clinical and/or research applications of TMS are numerous and multifaceted. A consistent number of different parameters, indeed, do allow wide-ranging assessments of the motor system both in terms of its functional/physiological features and pathologies as well. For instance, from a functional point of view, TMSs can be successfully used to study the existence of possible causal relationships between the stimulated area and related behavioral outcomes [3]; unquestionably, this is an extremely promising approach to study specific brain areas and related induced behaviors because it is possible to establish a direct link between the magnetic stimulation and the features of resulting behavior. From a clinical perspective, e.g., in the study of Parkinson’s disease, TMS can be successfully used to differentiate between idiopathic conditions and other forms characterized by the involvement of the corticospinal routes [3]. One aspect strictly related to TMSs that is still a matter of debate concerns the so-called “afferent inhibition”. A clear outline of afferent inhibition and its different characteristics can be found in an elegant review by Turco and Colleagues [4]. In brief, when a preliminary electric stimulus is delivered to a peripheral nerve, the amplitude of MEP elicited by TMS is reduced. Such a phenomenon is called “afferent inhibition” and can be classified in two different forms, on the basis of the interval length separating the stimulation of the nerve and the delivery of the TMS pulse [4]. If such an interval is relatively long (i.e., between 200 milliseconds and 1 s) it is called *long-latency afferent inhibition* (LAI) [4]; on the other hand, if the interval is relatively brief (i.e., between 20 and 50 milliseconds), it is called *short-latency afferent inhibition* (SAI) [4]. Although interesting models involving thalamocortical relays to the primary somatosensory cortex have been proposed (e.g., see [4]), neural mechanisms underlying LAI and SAI still remain a matter of debate. Therefore, not surprisingly, possible behavioral correlates of LAI and/or SAI do similarly remain a topic still scantly investigated/understood. In reality, the evaluation of LAI and SAI and their possible correlation with specific behavioral tasks could be an extremely promising direction of research because it could be able to unveil new approaches to understand neurological conditions characterized by movement disorders. Both LAI and SAI, indeed, could be profitably used as indicators to assess functional recovery in subjects with pathologic alterations of motor control [4].

In this context, it is possible to find the rationale of a recent interesting study concerning the identification of behavioral correlates of LAI and SAI by utilizing three different behavioral tasks [5], that is, Temporal Order Judgement test (TOJ), Grating Orientation test (GOT) and Purdue Pegboard Test (PPT). TOJ is a sensory task used to evaluate the temporal tactile acuity. In this test, the subject’s right hand is in a prone position and a device delivers tactile stimuli to two fingers. The participant must indicate the order of stimuli, i.e., which finger received the stimulus first. GOT is another sensory task used to study spatial tactile acuity. Here, the participant must indicate the order of specific stimuli, applied to the right index finger by means of a set of JVP domes (that is, small grating surfaces of different dimensions). Finally, PPT is a motor task employed to evaluate manual dexterity. In brief, the subject must insert with his right hand the higher possible number of small pegs in specific holes of a pegboard within a given time. The study, very well conducted, revealed a lack of any correlation between the magnitude of LAI or SAI and the three tasks mentioned above. Thus, on the basis of the available results, it has been concluded that TOJ, GOT and PPT cannot be used to evaluate behavioral correlates for afferent inhibition. However, as highlighted [5], three limitations could underlie these null findings: (i) a sample size not adequate for all the behavioral tests, (ii) the utilization of a single inter-stimulus interval (ISI) to evoke LAI and SAI respectively, and (iii) the assessment of possible relationships between afferent inhibition and behaviors, for each task, carried out by means of correlation indexes and not by assessing the contextual behavioral performance. Of course, a small sample size could be critical in the determinism of over- or underestimated data, and, obviously, the characteristics of the stimulation might also be quite important because it is not possible to exclude that, with different ISIs, different results could be found. That said, in my opinion, the most important among the three issues above-mentioned is the third, i.e., the evaluation of possible relationships between afferent inhibition and behavior, in each of the three tasks, in a non-contextualized fashion, exclusively on the basis of purely quantitative parameters. It is my contention that the possibility to describe a given behavior by means of hundreds, or even thousands, of numbers, does not imply the possibility to utilize those numbers to reconstruct the behavior in its comprehensiveness and, importantly, in its most intimate meanings and features. In other terms, we can describe a given behavioral component by using an enormous amount of numbers (latencies, frequencies, percent distributions, durations, etc.) but by doing so, each value will describe just a fragment of the original behavioral repertoire. For example, by studying a rodent in an open-field it is possible to *numerically quantify* several aspects of its behavioral performance: how much time the subject spends in the central or peripheral zones of the apparatus, the number of walking episodes, the latency of the first climbing, rearing or grooming, the duration of each of these components, etc. Just to name a few. By doing so, however, we will obtain, albeit numerous, only *simple information of unconnected behavioral elements*: fragments of what the *real* behavior of the subject actually is. Another approach, *to be considered not alternative but synergistic and additive to the quantitative evaluations*, is represented by the assessment of the relationships among the components of the behavioral repertoire in order to evaluate what is the real structure of the behavior, that is, *what actually the subject is really doing*. In this realm, the questions will no longer be “How many times does the subject walk” or “how long does each face-grooming episode last on average” but “*in what relationship is walking with face-grooming*” or “*how are they both related to sniffing*”. Importantly, following the administration/introduction of an independent variable, it will be possible to provide exhaustive answers concerning modifications in the *structure* and in the *organization* of the behavior. Following this line of reasoning, the next step is represented by a simple question: *how* do we describe these structural relationships?

A pupil of Konrad Lorenz (Nobel Prize in 1973, with Nikolaas Tinbergen and Karl von Frisch), Irenäus Eibl-Eibesfeldt, is one of the fathers of modern ethology. He was one of the first scientists to recognize the enormous importance of the temporal dimension in the study of behavior and the intrinsic difficulties of a behavioral analysis when such an aspect is taken into consideration. As underlined by Professor Eibl-Eibesfeldt, indeed, “*Behavior consists of patterns in time. Investigations of behavior deal with sequences that, in contrast to bodily characteristics, are not always visible*” [6]. In other terms, the study of behavior, in its most intimate structure, implicitly deals with time and, for this reason, behavioral dynamics are normally hidden and are not intuitively perceivable. More than a simple concept, the above-mentioned Eibl-Eibesfeldt idea can be considered a pivotal tenet in modern behavioral sciences. Not unexpectedly, given their features orbiting around elusiveness and inaccessibility, behavioral patterns have been particularly difficult to be studied for a long time. These difficulties have moved their steps around a multifactorial origin concerning, first of all, the chronic lack of an adequate formalized model of the patterns to detect; second, a similarly chronic lack of suitable detection algorithms; last but not least, the difficult implementation of these detection routines in specific software tools. These obstacles, over the last thirty years or so, in parallel with the extraordinary growth and development of personal computers and computational capabilities, have been gradually removed as a result of the introduction of a technique known as *T-pattern analysis* (TPA) [7,8,9,10,11,12,13,14,15,16,17,18,19]. By means of TPA, recurring patterns of events, impossible to detect, can be unveiled and analyzed. Simply stated, it is a multivariate approach able to detect the existence of statistically significant constraints among behavioral events within a given observation period of any length from the infinitesimally short to the extremely long (namely, from milliseconds to seconds, minutes, hours, days, etc.). In other terms, it does not matter how brief or long is the time observation window because the analysis follows exactly the same rules and principles. T-pattern detection can be performed by means of a software tool known as Theme and the outcomes of such a detection procedure are the so-called *T-patterns*. Simply stated, a T-pattern represents the detection of repetitive features of a behavior [9,10,11,13,14,17,18,19] and can be summarized using the following notation:

X1 ≈ dt1 X2 ≈ dt2 X3 ... Xi ≈ dti Xi + 1

in this expression, X terms, such as X1… X2… etc., do indicate the events belonging to a hypothetical T-pattern and ≈dt terms highlight the temporal distances separating these events in sequence. Accordingly, the term Xi ≈ dti Xi + 1 indicates that the event Xi is followed dti time units later by the following Xi + 1 event. Basically (Figure 1), a detection algorithm operating within a given time window encompassing a number of events (e.g., A, B, C, D...) compares the distribution of each pair of events (e.g., A and B) searching for a time interval so that the event A is followed by the event B, within that interval, more often than expected by mere chance. If such a circumstance, statistically verified, exists, A and B are by definition a first-level two-event T-pattern and indicated as (A B). In a second step, such a first-level (A B) T-pattern is used by the software algorithm to detect higher-order patterns, e.g., ((A B) C), ((A B) (C D)) … and so on. Such a process, running in a bottom-up fashion, runs up to any level and is completed when no more patterns are found.

Over the last two decades, TPA has been widely used by scientists from numerous research areas, such as computer science, psychology, psychiatry, physiology, sport science, biology, just to name a few. This approach has been able to offer an extraordinary level of detail in behavioral analyses, both in human and animal subjects as well, greatly beyond what the human eye can intuitively interpret by means of more conventional behavioral analyses.

For example, by means of such a technique, it has been possible to study route-tracing stereotypies in mice [20], the interaction between humans and artificial agents [21] or between human and animals [22], neuropsychiatric diseases [23,24,25,26], interactions between hormones and behavior [27], feeding behavior in rodents [28], the behavior in a model of Tourette’s syndrome [29] or Parkinson’s Disease [30], and anxiety-related behaviors in different rat strains [31,32,33,34,35,36,37,38,39,40]. Notably, TPA has been profitably used also to study movement and behavioral disorders both in human and non-human subjects (for a review, see [41]). As mentioned above, TPA should not be seen as a substitute approach to the more conventional quantitative analyses, but additive and synergistic. In fact, very frequently, the TPA has been able to reveal features of a given behavior otherwise invisible through simple analyses of frequencies, durations, etc. For instance, in our recent study concerning the impact of chronic nicotine exposure on the structure of anxiety-related behaviors in rats and the role of the lateral habenula [40], from our quantitative data, there was no observed interaction between nicotine treatment and lesion status. Such a result would have suggested that lateral habenula lesion had no role in the anxiety-related effects of nicotine; on the other hand, TPA has been able to reveal effects otherwise undetectable, that is, a complex modulatory role of the lateral habenula under chronic nicotine exposure [40]. Similar examples can be presented for the largest amount of studies applying TPA both in human and non-human subjects: actually, *TPA has been able to reveal behavioral features unnoticeable by means of quantitative analyses in the largest amount of studies that have utilized such an approach* [13,14,17,19]. Thus, concerning TOJ, GOT and PPT, it would be very interesting to perform the analysis on datasets obtained from recorded video files containing the behavior of each participant. The possibility to compare the behavior of subjects under different conditions, stimulation parameters, etc., could offer new insights concerning the behavioral correlates of afferent inhibition. Such an aspect implies the availability of the kind of data (technically called “T-data”) necessary to run the T-pattern detection process. These data, mandatorily: (i) must consist of *discrete events*, (ii) occurring within a *continuous observation period* and, (iii) on a *discrete scale* [10,15,18]. In addition, each sample must be stored in event log files. These files must simply contain the onset of each given behavior (and often also its conclusion, depending on the kind of annotation process performed) and the related time point. Notably, if samples are numerous (for instance, observations performed in a number of subjects and/or repeated instances in each subject) event log files can be concatenated and analyzed together. This is an issue to consider with extreme attention because a T-pattern may occur only one time in each sample but, at the same time, may be present in numerous samples. Finally, it is important to emphasize here that, on the basis of what is mentioned above, the analysis of the temporal structure essentially requires that everything occurring within the observation time window must be annotated with utmost precision and by highly trained observers. Normally, event log files required for T-pattern detection are obtained by means of multimedia coders, that is, software tools utilized to observe video files and annotate a given event performed by the subject and the related time point. Of course, event log files may also contain data extracted from other different sources. Theories, concepts and procedures underlying the detection and analysis of T-patterns can be found in our monographic works [14,19], in our reviews [13,17] and in numerous papers from the author of T-pattern detection and analysis, Prof. Magnus S. Magnusson [9,10,11,15,18].

## Figures and Tables

**Figure 1:**
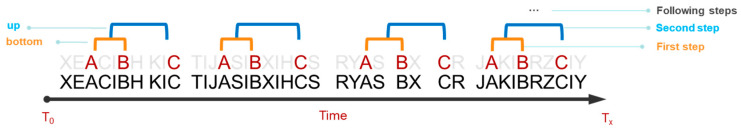
Hypothetical T-pattern consisting of three events during an observational period (T0–Tx) encompassing a total of 40 events (bold letters near the time axis). The ((A B) C) T-pattern becomes evident if all the remaining behavioral events are left out (grey letters). “First”, “second” and “following steps” indicate search runs carried out by Theme software following a bottom-up detection process.

## References

[1] Barker A.T., Jalinous R., Freeston I.L. (1985). Non-invasive magnetic stimulation of the human motor cortex. Lancet.

[2] Hallett M. (2000). Transcranial magnetic stimulation and the human brain. Nature.

[3] Rossini P.M., Rossi S. (2007). Transcranial magnetic stimulation: Diagnostic, therapeutic, and research potential. Neurology.

[4] Turco C.V., El-Sayes J., Savoie M.J., Fassett H.J., Locke M.B., Nelson A.J. (2018). Short- and long-latency afferent inhibition; uses, mechanisms and influencing factors. Brain Stimul..

[5] Turco C.V., Locke M.B., El-Sayes J., Tommerdahal M., Nelson A.J. (2018). Exploring Behavioral Correlates of Afferent Inhibition. Brain Sci..

[6] Eibl-Eibesfeldt I. (1970). Ethology: The Biology of Behavior.

[7] Blanchet A., Magnusson M.S. (1988). Processus cognitifs et programmation discursive dans l’entretien de recherche. Psychol. Française.

[8] Montagner H., Magnusson M., Casagrande C., Restoin A., Bel J.P., Hoang P.N.M., Ruiz V., Delcourt S., Gauffier G., Epoulet B. (1990). A new method for the study of behavioural organizers and interaction processes in infants. The first data. Psychiatr. L’Enfant.

[9] Magnusson M.S. (1996). Hidden Real-Time Patterns in Intra- and Inter-Individual Behavior: Description and Detection. Eur. J. Psychol. Assess..

[10] Magnusson M.S. (2000). Discovering hidden time patterns in behavior: T-patterns and their detection. Behav. Res. Methods Instrum. Comput..

[11] Magnusson M.S., Oller D.K., Griebel U. (2004). Repeated Patterns in Behavior and Other Biological Phenomena.

[12] Magnusson M.S. (2005). Understanding social interaction: Discovering hidden structure with model and algorithms. The Hidden Structure of Interaction: From Neurons to Culture Patterns.

[13] Casarrubea M., Jonsson G.K., Faulisi F., Sorbera F., Di Giovanni G., Benigno A., Crescimanno G., Magnusson M.S. (2015). T-pattern analysis for the study of temporal structure of animal and human behavior: A comprehensive review. J. Neurosci. Methods.

[14] Magnusson M.S., Burgoon J.K., Casarrubea M. (2016). T-pattern detection and analysis with THEME. Discovering Hidden Temporal Patterns in Behavior and Interaction.

[15] Magnusson M.S., Magnusson M.S., Burgoon J.K., Casarrubea M. (2016). Time and Self-Similar Structure in Behavior and Interactions: From Sequences to Symmetry and Fractals. Discovering Hidden Temporal Patterns in Behavior and Interaction.

[16] Magnusson M.S. (2017). Why Search for Hidden Repeated Temporal Behavior Patterns: T-Pattern Analysis with Theme. Int. J. Clin. Pharmacol. Pharmacother..

[17] Casarrubea M., Magnusson M.S., Anguera M.T., Jonsson G.K., Castañer M., Santangelo A., Palacino M., Aiello S., Faulisi F., Raso G. (2018). T-pattern detection and analysis for the discovery of hidden features of behaviour. J. Neurosci. Methods.

[18] Magnusson M.S. (2020). T-Patterns, external memory and mass-societies in proteins and humans: In an eye-blink the naked ape became a string-controlled citizen. Physiol. Behav..

[19] Casarrubea M., Di Giovanni G. (2020). Application of T-pattern Analysis in the study of the organization of behavior. Physiol. Behav..

[20] Bonasera S.J., Schenk A.K., Luxenberg E.J., Tecott L.H. (2008). A novel method for automatic quantification of psychostimulant-evoked route-tracing stereotypy: Application to Mus musculus. Psychopharmacology.

[21] Kerepesi A., Kubinyi E., Jonsson G.K., Magnusson M.S., Emiklósi A. (2006). Behavioural comparison of human-animal (dog) and human-robot (AIBO) interactions. Behav. Process..

[22] Kerepesi A., Jonsson G.K., Emiklósi Á., Topál J., Csányi V., Magnusson M.S. (2005). Detection of temporal patterns in dog-human interaction. Behav. Process..

[23] Lyon M., Kemp A.S. (2004). Increased temporal patterns in choice responding andaltered cognitive processes in schizophrenia and mania. Psychophamacology.

[24] Kemp A.S., Fillmore P.T., Lenjavi M.R., Lyon M., Chicz-DeMet A., Touchette P.E., Sandman C.A. (2008). Temporal patterns of self-injurious behavior correlate with stress hormone levels in the developmentally disabled. Psychiatry Res..

[25] Sandman C.A., Kemp A.S., Mabini C., Pincus D., Magnusson M. (2012). The role of self-injury in the organisation of behaviour. J. Intellect. Disabil. Res..

[26] Kemp A.S., Lenjavi M.R., Touchette P.E., Pincus D., Magnusson M.S., Sandman C.A. (2016). The Self-Organization of Self-Injurious Behavior as Revealed through Temporal Pattern Analyses. Discovering Hidden Temporal Patterns in Behavior and Interaction.

[27] Hirschenhauser K., Frigerio D., Grammer K., Magnusson M.S. (2002). Monthly Patterns of Testosterone and Behavior in Prospective Fathers. Horm. Behav..

[28] Casarrubea M., Aiello S., Di Giovanni G., Santangelo A., Palacino M., Crescimanno G. (2019). Combining Quantitative and Qualitative Data in the Study of Feeding Behavior in Male Wistar Rats. Front. Psychol..

[29] Santangelo A., Bortolato M., Mosher L.J., Crescimanno G., Di Giovanni G., Cassioli E., Ricca V., Casarrubea M. (2018). Behavioral fragmentation in the D1CT-7 mouse model of Tourette’s syndrome. CNS Neurosci. Ther..

[30] Casarrubea M., Di Giovanni G., Crescimanno G., Rosa I., Aiello S., Di Censo D., Ranieri B., Santangelo A., Busatta D., Cassioli E. (2019). Effects of Substantia Nigra pars compacta lesion on the behavioral sequencing in the 6-OHDA model of Parkinson’s disease. Behav. Brain Res..

[31] Casarrubea M., Sorbera F., Magnusson M., Crescimanno G. (2010). Temporal patterns analysis of rat behavior in hole-board. Behav. Brain Res..

[32] Casarrubea M., Sorbera F., Magnusson M.S., Crescimanno G. (2011). T-pattern analysis of diazepam-induced modifications on the temporal organization of rat behavioral response to anxiety in hole board. Psychopharmacology.

[33] Casarrubea M., Roy V., Sorbera F., Magnusson M.S., Santangelo A., Arabo A., Crescimanno G. (2013). Significant divergences between the temporal structure of the behavior in Wistar and in the spontaneously more anxious DA/Han strain of rats tested in elevated plus maze. Behav. Brain Res..

[34] Casarrubea M., Roy V., Sorbera F., Magnusson M.S., Santangelo A., Arabo A., Crescimanno G. (2013). Temporal structure of the rat’s behavior in elevated plus maze test. Behav. Brain Res..

[35] Casarrubea M., Magnusson M.S., Roy V., Arabo A., Sorbera F., Santangelo A., Faulisi F., Crescimanno G. (2014). Multivariate temporal pattern analysis applied to the study of rat behavior in the elevated plus maze: Methodological and conceptual highlights. J. Neurosci. Methods.

[36] Casarrubea M., Cancemi D., Cudia A., Cardaci M., Sorbera F., Faulisi F., Magnusson M.S., Crescimanno G. (2015). Application of multivariate T-pattern analysis in the study of social interaction in rats. J. Biol. Res..

[37] Casarrubea M., Davies C., Faulisi F., Pierucci M., Colangeli R., Partridge L., Chambers S., Cassar D., Valentino M., Muscat R. (2015). Acute nicotine induces anxiety and disrupts temporal pattern organization of rat exploratory behavior in hole-board: A potential role for the lateral habenula. Front. Cell. Neurosci..

[38] Casarrubea M., Faulisi F., Caternicchia F., Santangelo A., Di Giovanni G., Benigno A., Magnusson M.S., Crescimanno G. (2016). Temporal patterns of rat behaviour in the central platform of the elevated plus maze. Comparative analysis between male subjects of strains with different basal levels of emotionality. J. Neurosci. Methods.

[39] Casarrubea M., Pierucci M., Aiello S., Cassar D., Deidda G., Crescimanno G., Di Giovanni G. (2020). Effects of chronic nicotine on the temporal structure of anxiety-related behavior in rats tested in hole-board. Prog. Neuro-Psychopharmacol. Biol. Psychiatry.

[40] Casarrubea M., Davies C., Pierucci M., Colangeli R., Deidda G., Santangelo A., Aiello S., Crescimanno G., Di Giovanni G. (2021). The impact of chronic daily nicotine exposure and its overnight withdrawal on the structure of anxiety-related behaviors in rats: Role of the lateral Habenula. Prog. Neuro-Psychopharmacol. Biol. Psychiatry.

[41] Aiello S., Crescimanno G., Di Giovanni G., Casarrubea M. (2020). T-patterns in the study of movement and behavioral disorders. Physiol. Behav..

